# ML-based validation of experimental randomization in learning games

**DOI:** 10.3389/frai.2025.1541087

**Published:** 2025-10-30

**Authors:** Pei-Hsuan Hsieh

**Affiliations:** ^1^Department of Computer Science, College of Informatics, National Chengchi University, Taipei, Taiwan; ^2^College of Education, National Chengchi University, Taipei, Taiwan

**Keywords:** randomization, experimental design, sample assignment, scenarios, machine learning (ML) model, classification performance, learning game

## Abstract

Randomization is a standard method in experimental research, yet its validity is not always guaranteed. This study introduces machine learning (ML) models as supplementary tools for validating participant randomization. A learning direction game with dichotomized scenarios was introduced, and both supervised and unsupervised ML models were evaluated on a binary classification task. Supervised models (logistic regression, decision tree, and support vector machine) achieved the highest accuracy of 87% after adding synthetic data to enlarge the sample size, while unsupervised models (k-means, k-nearest neighbors, and ANN—artificial neural networks) performed less effectively. The ANN model, in particular, showed overfitting, even with synthetic data. Feature importance analysis further revealed predictors of assignment bias. These findings support the proposed methodology for detecting randomization patterns; however, its effectiveness is influenced by sample size and experimental design complexity. Future studies should apply this approach with caution and further examine its applicability across diverse experimental designs.

## Introduction

When conducting experimental research, randomization of research participants is the most commonly used method, typically followed by a series of comparisons between at least two different groups of participants (Campbell and Stanley, 2015). A two-group comparison can be referred to as dichotomization (DeCoster et al., 2009; MacCallum et al., 2002). Randomization can be based on participants’ demographic differences, such as gender (e.g., Chung and Chang, 2017) or game experience levels (e.g., Procci et al., 2013). From the perspective of game design in educational contexts, students’ learning performance can also be compared after assigning them to play different types of games (e.g., two- versus three-dimensional; Yilmaz and Cagiltay, 2016). A fair comparison in such a dichotomized setting can be expected if participants are appropriately assigned to one of the two game types in the experiment.

Machine learning (ML) models are broadly categorized into supervised (e.g., logistic regression, support vector machines) and unsupervised approaches (e.g., k-means clustering, artificial neural networks Verma et al., 2022). Both types are particularly valuable for studying game behavior in educational settings. For example, ML was applied to enhance the analysis of game-based learning data and to highlight the value of game behaviors (e.g., Järvelä et al., 2020; Luan and Tsai, 2021). In experimental research, ML can serve as a novel approach to detecting randomization flaws, complementing conventional balance tests. While statistical methods such as the *t*-test and chi-square test are commonly used to examine the validity of randomization, they may be limited in addressing fluid, high-dimensional, and nonlinear relationships among predictive factors (Choubineh et al., 2023). ML, by contrast, enables the detection of complex patterns across all data points in an experimental study (Verma et al., 2022). This research preliminarily explores the capability of ML models to classify well-randomized, dichotomized sample assignments. Successful classification is assumed to provide evidence of the validity of these assignments within the experimental design.

As researchers claim that their sample assignments are well-randomized, the classification performance of ML, measured by accuracy rate and influenced by implementation settings, is expected to surpass a satisfactory threshold (e.g., 60% or higher; Zhang et al., 2017). In a fully randomized experiment, ML models can evaluate the effectiveness of dichotomization in sample randomization. A high accuracy rate in classifying samples within a dichotomized experimental design reinforces the validity and reliability of claims regarding the random assignment process. In addition, verification through ML not only supports researchers’ claims but also enhances their academic credibility by demonstrating robust sample randomization in experimental studies.

This study raises the research question: Can ML models serve as a methodological validation tool to enhance researchers’ accountability in claiming proper randomization in experiments? It is hypothesized that supervised ML models will outperform unsupervised ones in classifying sample assignments, while feature importance analysis from unsupervised ML models will reveal key predictors of assignment bias. Both approaches can provide insights into the effectiveness of small sample sizes and within-group experimental designs. Unlike prior studies focusing on game analytics, the novelty of this study lies in validating the capability of both supervised and unsupervised ML models to examine randomization in experimental assignments.

The following sections begin with a review of psychology research literature on randomization in sample assignment, followed by a discussion of the classification capabilities of ML models. To address the proposed research question, a learning game featuring two distinct scenarios was introduced. A detailed description of the recruitment plan and methodology, including randomization procedures, data collection, implementation of various ML models, and analysis methods, is provided. The results of employing these ML models are presented and thoroughly discussed. Finally, the capability of ML models to enhance research validity and reliability in experimental studies, as well as to analyze various behaviors in learning games, is explored.

### Related work

#### Randomization approaches in experimental design

Randomization is widely regarded as one of the most effective solutions for experimental design in psychological research (Campbell and Stanley, 2015; Pirlott and MacKinnon, 2016). Randomization can effectively neutralize participant characteristics (e.g., age and gender) and ensure a representative selection of participants from the research context (e.g., Chung and Chang, 2017; Pan and Ke, 2023; Procci et al., 2013; Puolakanaho et al., 2020). Given the limitations of the research context, incorporating a control group becomes essential to rule out confounding factors (e.g., individual differences in personality and prior knowledge) within the experimental group, a design known as a randomized controlled design or trial (e.g., Puolakanaho et al., 2020). A within-subject design can also be used to address confounding factors among research participants (Cohen and Staub, 2015; Brauer and Curtin, 2018; Montoya, 2023). In this approach, participants’ characteristics can later serve as control variables during the data analysis stage to enhance the robustness of the findings (Campbell and Stanley, 2015; Bernerth and Aguinis, 2016).

In addition, sampling designs that consider population characteristics are also an effective approach to preventing prominent bias and confounding effects in the study context (Levy and Lemeshow, 2013; Lohr, 2021). This research method is crucial and widely used in large-scale educational studies, such as the Program for International Student Assessment (Organization for Economic Co-operation and Development, 2014) and Trends in International Mathematics and Science Study TIMSS (Martin et al., 2016). The sampling weights provided by the experts of these large-scale studies can be used by researchers who focus on providing meaningful suggestions for educational, political, or healthcare policies to generate generalizable findings for the broader population (Arıkan et al., 2020; Ertl et al., 2020; Laukaityte and Wiberg, 2018; Meinck, 2015; Rust, 2014). Practitioner researchers can also model the sampling design using various statistical analyses (e.g., multilevel analysis) to identify the impact of the factors considered in the sampling design (Chiu et al., 2022; Laukaityte and Wiberg, 2018).

Independent researchers in some fields (e.g., education, neurology, psychology) often use small sample sizes to examine their theories (Francis et al., 2010; Lakens, 2022; Vasileiou et al., 2018). The within-subject design is typically the most appropriate and convenient choice, offering higher statistical power (Lakens, 2022) despite potential carryover effects (e.g., fatigue and order effects, Montoya, 2023). A limitation is that researchers may struggle to determine the appropriateness of this design compared with the randomization design.

#### Classification performance of machine learning (ML) models

Machine learning models have gradually been used to analyze large datasets. Data come from online learning platforms, such as ASSISTment (Feng and Heffernan, 2007) and large-scale datasets, such as PISA (Organization for Economic Co-operation and Development, 2014) and TIMSS (Martin et al., 2016). Learning analytics and educational data mining are the two general terms for research utilizing machine learning (ML) or artificial intelligence (AI) models to advance ideals in education or psychology, such as personalized learning or adaptive instruction (Heffernan and Heffernan, 2014; Khan and Ghosh, 2021; Zotou et al., 2020). For example, affective computing can detect students’ affective status when engaging in learning or problem-solving using a computer (Baker et al., 2010; Järvelä et al., 2020).

ML models include two main categories: Supervised and unsupervised learning (Verma et al., 2022). Supervised learning relies on labeled data. Dichotomous data (e.g., correct vs. incorrect answers or gameplay sequences in this study) appear to be the basic, widely used criterion in ML (Goretzko and Bühner, 2022; Yeung and Fernandes, 2022). In the supervised learning model, the dataset was split into two parts: training and testing, with a split ratio of 80/20 (Bichri et al., 2024). With human-proved criteria, supervised learning is effective in training models by using labeled data to fit the final practical use (Alloghani et al., 2019; Verma et al., 2022). In other words, supervised learning increases ecological validity for the human world. Contrarily, without knowing any ground truth (i.e., labeled data), the unsupervised learning model aimed to identify patterns in the data points (Alloghani et al., 2019). Unsupervised learning viewed as more exploratory and data-driven (not based on criteria or theories) can still be useful in model training as long as the pattern could be found from unlabeled data. Thus, unsupervised learning models are used to identify unknown categories, similar to exploratory factor analysis (EFA) in psychology and education (Cox et al., 2020). The main difference between supervised and unsupervised learning models is whether labeled data are used during the training process (Verma et al., 2022). This distinction allows for the potential evaluation of the effectiveness of a within-subject experimental design, which typically involves small sample sizes and only a few factors (e.g., gender), in a manner similar to that of a randomized design. To be noted, the results obtained by unsupervised learning usually need further validation (Xie et al., 2020; Zimmermann, 2020), just like the confirmation factor analysis to validate the results obtained by EFA.

#### Theoretical foundations for employing ML models in randomization validation

There are several ways to check whether samples have been randomly assigned into groups in an experiment (Bruhn and McKenzie, 2009; Campbell and Stanley, 2015). Common approaches include examining descriptive statics (e.g., mean, standard deviation, distribution shape via histograms or boxplots), running statistical tests (e.g., *t*-test, ANOVA, chi-square test), and creating a balance check table to list all relevant pre-treatment covariates, group means, *p*-values from statistical tests, and mean differences. However, traditional statistical tests are not always sufficient when sample characteristics are fluid and unstable over time (e.g., emotion, political inclination), when demographics involve high-dimensional data (e.g., brain neuron connectivity), or when unpredictable circumstances introduce nonlinear relationships during the experiment (e.g., bad weather, participant tardiness). While such complexities may be difficult for researchers to detect, various ML models can help uncover these patterns and provide supporting evidence (e.g., Choubineh et al., 2023). Researchers are encouraged to make effective use of ML techniques to generate classification results. These results can then be used to assess whether standardized randomization has reached an acceptable threshold (e.g., 60% or higher; Zhang et al., 2017).

In this exploratory research, ML models are employed to detect patterns or predictive relationships among data points collected from an experiment designed to undergo proper randomization. ML classifiers are expected to perform beyond chance levels (e.g., ~50% accuracy in a binary classification task). Grounded in mathematical principles, both classification and clustering algorithms can be used to evaluate whether the claim of dichotomized, randomly assigned samples is valid. If randomization is achieved, the performance of supervised ML models on classification tasks is expected to reach a satisfactory level, defined here as standardized ML performance metrics meeting a benchmark accuracy rate of at least 60%, with measures such as accuracy, precision, recall, and F1-score used to assess performance (Zhang et al., 2017). It should be noted that AUC-ROC and support were not included in this study, as the focus is on validating whether randomization was achieved, rather than on ranking quality (as in AUC-ROC) or the number of supporting cases in a binary classification (Verma et al., 2022).

Supervised ML models are expected to perform effectively in classifying participants into their assigned scenarios. Unsupervised ML models, by contrast, may identify patterns through clustering that appear to improve classification performance but risk overlooking the validity of the underlying claim. In other words, assignment bias may be detected through unsupervised ML models. Large differences in feature values across cluster centroids can indicate that specific features (e.g., demographic attributes) are driving group separation. Conversely, if substantial centroid differences emerge in unexpected features (e.g., attributes unrelated to participants), this may provide evidence of systematic assignment bias.

### Methodologies

**Figure 1:**
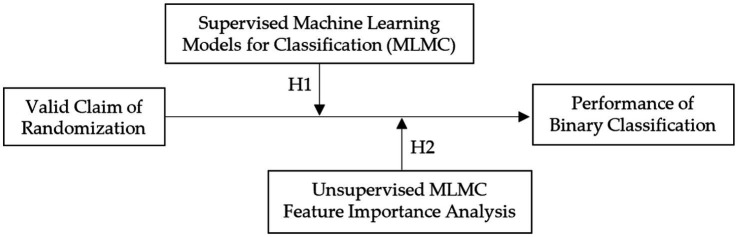
Research model.

This exploratory study implements a fully randomized experiment with two scenarios. Two hypotheses are proposed, drawing on ML classification and clustering algorithms grounded in mathematical principles. Supervised ML is applied to labeled data (e.g., group assignments), enabling the model to map features to known labels. By contrast, unsupervised ML is employed to uncover latent structures or groupings in unlabeled data (e.g., clustering) (Figure 1). It is hypothesized that supervised ML will perform effectively under randomized conditions, and that clustering within unsupervised ML may yield even higher apparent classification performance (H1).

In unsupervised ML, large differences in feature values across cluster centroids may indicate that a given feature (e.g., a demographic attribute of participants) contributes significantly to group separation. Conversely, if such differences emerge in features where no separation is expected (e.g., attributes unrelated to participants), this may signal assignment bias (H2). By employing both classification and clustering algorithms, the proposed hypotheses aim to evaluate model performance under conditions of valid randomization.

*H1*: Supervised MLs achieve higher accuracy than unsupervised ones in detecting randomization flaws.

*H2*: Feature importance analysis reveals key predictors of assignment bias.

### An experiment of learning direction game

**Figure 2:**
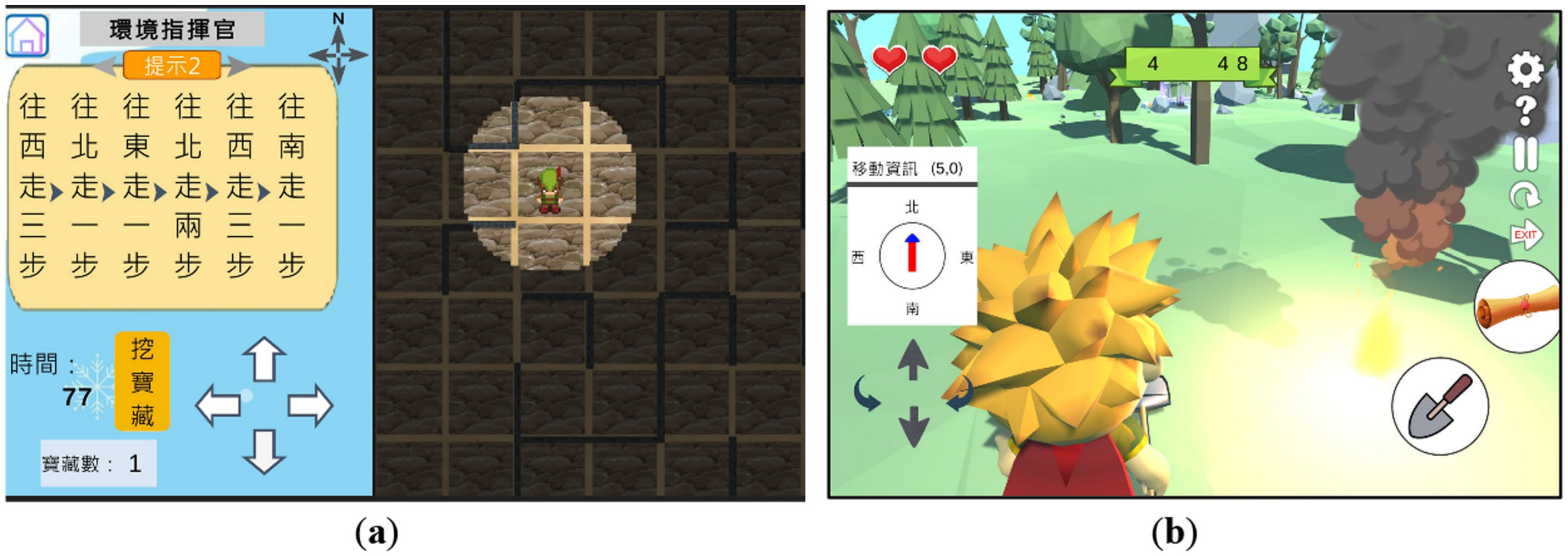
Learning direction game experimental scenarios designed in: **(a)** 2D and **(b)** 3D interfaces. This game was developed as part of the funded project (NSTC 110-2511-H-004-001-MY3).

This study employed *Utility* to develop a learning game with two distinct difficulty levels, as two scenarios used to test learning outcomes in the implementation of a fully randomized experiment. In the first scenario, participants used a 2D interface to guide a virtual agent through eight mazes, sequentially locating eight treasures (Figure 2a). In the second scenario, participants used a 3D interface to assist a different virtual agent in finding eight treasures by following assigned directions (Figure 2b). Both scenarios incorporated the same mathematical learning concept, i.e., the NSEW directional system, with a compass continuously displayed on the interface. Participants required some adaptation to orient themselves within the environment before gameplay. To enhance engagement, participants were encouraged to imagine themselves as the virtual agents while playing.

Each scenario included a time constraint: 100 s in the 2D interface and 240 s in the 3D interface. Participants’ performance was evaluated based on scores, with bonus points awarded for task completion within the allotted time. The maximum achievable score was 36 points in the 2D interface (eight treasures) and 80 points in the 3D interface (eight treasures).

### Participants

An equal number of female and male undergraduates (all aged 20 years) were recruited to minimize potential gender effects on learning performance in the game-based context. Participants were recruited from humanities-related disciplines (e.g., linguistics, philosophy, sociology), ensuring that their coursework did not require the use of information technologies. Prior to the experiment, a brief survey was administered to assess participants’ weekly gameplay frequency. In total, 12 participants (six female, six male) were recruited and randomly assigned to one of the two experimental scenarios. Randomization ensured balance across groups with respect to gameplay frequency: each group included two non-gamers, one seldom player, one occasional player, and two frequent players.

### Data collection

All participants were instructed to collect as many treasures as possible in each interface of their assigned treatment. In addition to the total scores obtained at the end of the game, task completion time each interface and the time required to collect each treasure were recorded. As summarized in Table 1, a total of 22 data points were collected for each participant across the two interfaces. For subsequent analyses, all variables except gender were normalized using ratio-based transformations to a 0–1 scale, ensuring a consistent basis for comparing the performance of different machine learning models.

**Table 1:** All data points collected in each interface.

Data types	Data points
Demographics	No. 1: Gender No. 2: Gameplay frequency
Learning scores	No. 3: Total scores obtained in 2D interface No. 4: Total scores obtained in 3D interface No. 5 ~ 12: Scores obtained from eight tasks in 2D interface No. 13 ~ 20: Scores obtained from eight tasks in 3D interface
Gameplay time usage	No. 21: Total playing time in seconds in 2D interface No. 22: Total playing time in seconds in 3D interface

### Experimental procedures

To ensure the validity of randomized sample assignment, an equal number of female and male participants with comparable gameplay frequency levels from humanities-related fields were evenly distributed across two treatment groups. In Treatment 1 (labeled 2D3D), participants played the direction learning game starting with the 2D interface, followed by the 3D interface. In Treatment 2 (labeled 3D2D), the sequence was reversed, with participants starting on the 3D interface and then moving to the 2D interface.

**Figure 3:**
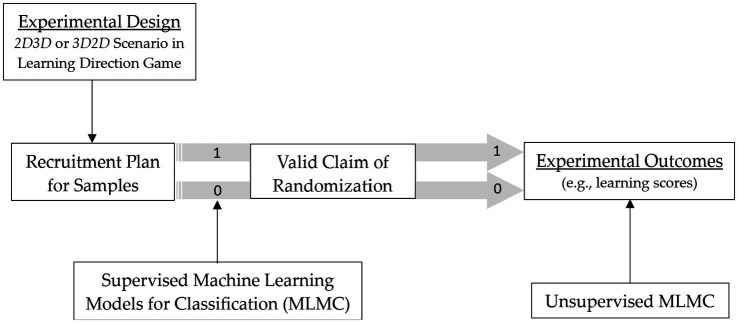
Experimental procedures.

Upon completion of the assigned sequence, data were collected on participants’ task completion times (speed in finding each treasure) and learning scores. As illustrated in Figure 3, both supervised and unsupervised ML models were developed using the dichotomized treatment assignments. In the supervised approach, the randomization sequence (2D3D vs. 3D2D) was explicitly provided as labels to facilitate learning. In contrast, unsupervised models attempted to recover the sequence structure directly from the experimental data. Binary classification performance was then evaluated to assess the extent to which ML models could validate the randomized experimental design.

To ensure consistency in comparison, the treatment assignment of each participant (i.e., group allocation by gender and gameplay frequency; see No. 1 ~ 2 in Table 1) was incorporated into the ML process. Performance scores (No. 3 ~ 20 in Table 1) and task completion times (No. 21 ~ 22 in Table 1) were split into training and test sets (80/20) for supervised model development, whereas in unsupervised model development these were treated as features. Because participants were randomly assigned according to the recruitment plan, which balanced gender and gameplay frequency across groups, the models could learn from both the equilibrium of group assignment and performance-related datapoints. To increase the effective sample size, synthetic data were generated by introducing random noise into existing data points.

### Data analyses

According to Table 1, the participants’ demographics were clearly reported in the recruitment plan. The analyses were first conducted to compare differences in the participants’ learning scores and the time spent playing the game between the two groups. Descriptive analyses were performed using the original scores, providing the mean and standard deviation. An independent samples *t*-test was then conducted to determine which group had significantly higher learning scores. All data points, except for gender, were normalized for later analyses.

Supervised and unsupervised machine learning (ML) models were used to test the proposed hypotheses. These two types of models differ based on the presence of labels in the dataset (Verma et al., 2022). Hypothesis testing referred to the ML performance on a binary classification task (Bichri et al., 2024). Model performance evaluation was based on standard classification metrics, including accuracy, precision, recall, and F1-score (Luan and Tsai, 2021; Zhang et al., 2017). All metrics were expressed in percentage format to enable direct comparison of classification performance across models. Based on the literature, satisfactory learning performance in the classification task was defined as achieving metrics that exceeded a passing threshold of 60% (Bichri et al., 2024; Zhang et al., 2017), even though higher thresholds may be needed for robust validation. Notably, this study used Jupyter Notebook 6.5.4 and Python 3 to run all models. All models were expected to perform well by accurately classifying the samples into two groups (i.e., 2D3D vs. 3D2D), based on the data points listed in Table 1.

Commonly-used ML models were selected to test the hypotheses (Alloghani et al., 2019; Luan and Tsai, 2021; Verma et al., 2022). The representative supervised learning models employed in this study were logistic regression, decision tree, and support vector machine (SVM). The supervised learning models were trained and tested using an 80/20 split ratio (Bichri et al., 2024). Except for logistic regression, decision tree and support vector machine (SVM) are considered non-parametric algorithms. Logistic regression is a statistical linear model in which a coefficient is estimated for each variable. Decision trees recursively partition the feature space using a tree-like structure, consisting of root, parent, child, and leaf nodes; pruning may be applied when necessary to avoid overfitting (Alloghani et al., 2019). In contrast, SVM constructs optimal separating hyperplanes to maximize the margin between classes without assuming a specific data distribution (Prastyo et al., 2020). By incorporating kernel functions, SVM can transform a non-linear dataset into a higher-dimensional feature space, enabling the construction of a linear separation boundary and potentially improving performance compared to the basic linear SVM model. In addition, model performance varied depending on the values of *random_state* and other parameter settings (e.g., depth of tree, kernel type), reflecting the sensitivity of decision trees and SVM to initialization and hyperparameter choices.

The representative unsupervised learning models used in this study were k-means, k-nearest neighbor (KNN), and artificial neural networks (ANN). K-means is a centroid-based algorithm (Alloghani et al., 2019). The numbers of clusters could be decided and then tested to determine whether the highest accuracy rate had been achieved. The value for each cluster center could be obtained. The value was a mean score representing the importance of the data point. A random seed could be provided to ensure the output was stable in each run of the learning process for the model. KNN is also a non-parametric algorithm, meaning it does not involve model learning or make assumptions about the data distribution (Luan and Tsai, 2021). It operates as a supervised classification model. In the unsupervised learning phase, the only step is to assign the number of neighbors for each sample in the training dataset. However, when the data is labeled, the model can make accurate classification decisions and perform well on classification tasks. Finally, ANN can be used in both supervised and unsupervised learning contexts. This model extracts statistical properties or data features from the training dataset to inform learning. There are various approaches to running an ANN in an unsupervised manner. For example, an autoencoder can be used to help the model learn from vectorized data points (Song et al., 2013). A clustering algorithm can also be used to the hidden layers of the network to identify patterns and groupings within the data.

## Results

Participants’ raw gameplay scores and completion time spent were first analyzed to evaluate their performance. Various supervised and unsupervised machine learning (ML) models were then trained using normalized data points and gender information collected from the randomized sample assignment in the experiment. Finally, the proposed hypotheses were tested based on the classification performance of each model.

### Learning scores and time usage in gameplay experiment

The 2D3D group achieved higher scores than the 3D2D group when playing the learning direction games on the 2D or 3D interfaces (Table 2). However, the difference in scores between the two groups was not statistically significant (only in 2D: *t* = 0.870, *p* = 0.413; only in 3D: *t* = 0.842, *p* = 0.612). In addition, the 2D3D group spent less time playing games on the 2D and 3D interfaces compared to the 3D2D group. To be noted, this comparison result is helpful to the machine accurately classifying samples in the follow-up model development and learning processes.

**Table 2:** Differences between two treatments in learning performance and time use.

Participation number	Biological gender	Game frequency*	2D Gameplay time usage (100 s)	2D Learning scores (36/36)	3D Gameplay time usage (240 s)	3D Learning scores (80/80)
2D3D Group (average scores in 2D: 19.67/36, s.d. = 13.09; average scores in 3D: 53.33/80, s.d. = 35.02)						
001	Female	1	100	10	240	60
002	Female	3	100	15	240	0
003	Female	2	100	6	191	80
007	Male	1	100	15	230	80
008	Male	4	74	36	221	80
009	Male	4	96	36	240	20
3D2D Group (average scores in 2D: 13.86/36, s.d. = 11.54; average scores in 3D: 50.00/80, s.d. = 23.80)						
004	Female	4	100	0	216	80
005	Female	1	100	28	240	30
006	Female	2	100	21	240	40
010	Male	3	100	21	234	80
011	Male	1	100	6	240	20
012	Male	4	100	21	240	40

*1 = almost none, 2 = a few times or about an hour per week, 3 = not often, 4 = often (more than three times or about 3 h per week).

### Machine learning model performance on binary classification

This section outlines the approach used to implement each ML model, followed by detailed explanations of data entries and data processing procedures. Default parameter values provided by the original models were used to ensure the stability of the learning procedures. For instance, the *random_state* parameter in the Python *Scikit-learn* library was set to an integer based on the data size for certain machine learning models (e.g., decision tree, k-means,). Finally, the performance metrics were reported to evaluate each model’s performance on the binary classification tasks in this study. Table 3 presents a comprehensive comparison of the results across all machine learning models.

**Table 3:** Performance metrics achieved by different machine learning models.

Machine learning models	A	P	R	F	Random state values and parameters used
Supervised					
Logistic regression (after adding synthetic data)	0.67 (0.80)	0.83 (0.87)	0.67 (0.80)	0.67 (0.80)	Train: test = 0.8:0.2, random_state = 114 Solver = lbfgs
Decision tree (after adding synthetic data)	0.67 (0.80)	0.44 (0.87)	0.67 (0.80)	0.53 (0.80)	Train: test = 0.8:0.2, random_state = 143 Depth of tree = 2
SVM (after adding synthetic data)	0.67 (0.80)	0.83 (0.87)	0.67 (0.80)	0.67 (0.80)	Train: test = 0.8:0.2, random_state = 114 Kernel = linear
Unsupervised					
K-Means (after adding synthetic data)	0.58 (0.58)	0.59 (0.59)	0.58 (0.58)	0.58 (0.58)	Cluster = 2, random_state = 1
KNN (after adding synthetic data)	0.67 (0.67)	0.83 (0.83)	0.67 (0.67)	0.67 (0.67)	Neighbor = 1, random_state = 1
ANN (after adding synthetic data)	0.67 (0.67)	0.44 (1.00)	0.67 (0.67)	0.53 (0.80)	activation and optimizer, random_state = 3 (random_state = 2)

A, accuracy rate; P, precision; R, recall; F, F1-score.

### Supervised learning models

In the supervised learning model, the training dataset was labeled to help the machine identify which samples were assigned to the 2D3D or 3D2D groups. The model’s performance on the binary classification task was then evaluated using the testing dataset. It is worth noting that the sample size in this study was small. Consequently, the data split into training and testing was conducted in a conventional manner, and the learning performance may be as high as reported in the literature.

#### Logistic regression

This model was used to classify samples, represented by 22 data points (Table 1), into the two treatment groups (2D3D and 3D2D). Using an 80/20 training-testing split and a *random_state* value of 114, the model achieved an accuracy of 67%. The solver parameter was set to its default (*lbfgs*), and alternative solvers (*liblinear*, *newton-cg*, *sag*, *saga*) produced identical results. However, when *random_state* values below or above 114 were applied, accuracy decreased substantially, in some cases dropping to 33% or even 0%. Following the incorporation of synthetic data, model performance improved, with accuracy reaching a maximum of 80% under the same parameter settings. Moreover, precision, recall, and F1-score each exceeded 80%, indicating that the model not only achieved higher overall accuracy but also maintained balanced and reliable classification performance across evaluation metrics.

#### Decision tree

In this study, the 22 data points were used as input features to predict the treatment groups (2D3D vs. 3D2D) by learning simple decision rules. The decision tree model consistently achieved a maximum accuracy of 67% with an 80/20 training–testing split and a *random_state* value of 143, which was higher than the value observed in the logistic regression model. The tree depth was set to the default configuration, allowing the tree to expand fully, while the default *random_state* parameter resulted in trees being built differently in each iteration. Notably, when *random_state* values lower or higher than 145 were applied during sample splitting, accuracy dropped sharply, reaching as low as 33% or even 0%. After the inclusion of synthetic data, model performance improved, with accuracy reaching 80% when the tree depth was restricted to 2 while keeping other parameters unchanged. Under these conditions, precision, recall, and F1-score also exceeded 80%, indicating improved and balanced classification performance.

#### Support vector machine (SVM)

The model was trained with an 80/20 training–testing split and a *random_state* value of 114. A *linear* kernel function was applied instead of the default *rbf* (radial basis function) kernel, resulting in an accuracy of 67%. Alternative kernels such as *poly* and *sigmoid* produced substantially lower accuracy. After adding the synthetic data, accuracy improved to 80% under the same parameter setting, with precision, recall, and F1-scores also exceeding 80%. However, when the *rbf* or *poly* kernels were used, the model achieved 100% accuracy, indicating potential overfitting. By contrast, the *sigmoid* kernel achieved only 40% accuracy, reflecting poor performance.

### Unsupervised learning models

The ground truth refers to the actual classification of the samples in this study, which is also known as the target. To predict the target accurately, the model learned from the given data points, which were treated as vector features. In other words, even though the dataset was not labeled, the model was still able to learn effectively based on the features during the learning process.

#### K-means

All 22 data points were used to predict participants’ gameplay sequence assignments by clustering the samples into two groups. The Elbow Method plot also confirmed that two clusters provided the best fit, consistent with the original random sample assignment (2D3D vs. 3D2D). The highest accuracy rate (58%) was achieved with *random_state = 1*. Increasing the *random_state* value did not improve accuracy. Other performance metrics included precision (59%), recall (58%), and F1-score (58%).

Feature importance analysis showed that the most influential variable was the total time spent collecting all eight treasures in the 3D game (No. 22 in Table 1). Gender and gameplay frequency were not identified as key predictors of assignment, although gender contributed slightly more than gameplay frequency. After incorporating synthetic data, results were reproduced consistently. In addition, Silhouette scores ranged from 0.165 to 0.410, suggesting that participants fit reasonably well within their assigned clusters compared to alternative cluster assignments (Alloghani et al., 2019; Prastyo et al., 2020). No evidence of overfitting was detected.

#### K-nearest neighbor (KNN)

Given the small sample size, a lower number of neighbors was deemed appropriate. Accordingly, the number of neighbors was set to 1 with *random_state = 1*, resulting in an accuracy rate of 67%, recall and F1-scores of 67%, and a higher precision score of 83%. These values were consistent across both the training and testing datasets. However, when the number of neighbors was increased to 2 or 3, accuracy dropped to 33%. Although using more than 4 neighbors occasionally achieve 67% accuracy, this was not considered reasonable given the limited sample size.

Since KNN is not a tree-based model (e.g., decision tree or random forest) and lacks built-in feature importance measures (unlike *RandomForestClassifier* or *GradientBoostingClassifier*) (Pudjihartono et al., 2022), feature relevance was examined through statistical methods, including chi-square tests, *f_classif*, and RFE (Recursive Feature Elimination). These analyses identified the following important features: (1) the time spent hunting the sixth treasure in the 3D game (No. 18 in Table 1), (2) the total time spent hunting all treasures in the 2D game (No. 21), and (3) the total scores earned in the 2D game (No. 3). Similar findings were obtained for gender and gameplay frequency in terms of their relatively low importance. After incorporating synthetic data, the same results were reproduced, confirming the robustness of the findings.

#### Artificial neural networks (ANN)

Since ANN can be applied in both supervised and unsupervised learning contexts, this study implemented both approaches by encoding participants’ gameplay scores and completion time collected from the 2D and 3D games, using two hidden layers. To mitigate overfitting, where the model fits the training data perfectly but generalizes poorly to the test data (Bejani and Ghatee, 2021), two activation functions were applied, i.e., *relu* (layer dense = 4) followed by *sigmoid* (layer dense = 1).

Hyperparameter tuning was then conducted using the *keras-tuner* library. The *ADAM* optimizer and categorical *cross-entropy loss* function were employed during training to minimize loss (Ghosh and Gupta, 2023). In addition, *GridSearch* was used to explore combinations of hyperparameters, limited to 10 trials (as overfitting was observed with more than 10 trials given the dataset size). Under these conditions, the ANN achieved its best accuracy of 67%, with *random_state = 3* for training–testing splitting and an optimal learning rate of 0.0001. Other metrics were also obtained but did not exceed 67%. In addition, feature relevance was examined with multiple statistical tests. The chi-square test identified the total scores participants received in the 2D game (No. 3, Table 1) as an important predictor. The *f_classif* and *RFE* tests highlighted the total time spent hunting all treasures in the 2D game (No. 21) as important. Gender and gameplay frequency were consistently found to have relatively low importance.

After synthetic data were incorporated, accuracy and recall remained unchanged with a lower *random_state*. The F1-score increased to 80%, while precision rose to 100%, indicating potential overfitting caused by false positives being misclassified. This suggests that the trained ANN may incorrectly classify well-randomized data as flawed. Nevertheless, across all three tests, the total 2D game score (No. 3) was consistently identified as an important feature in binary classification.

## Discussion

Randomization is critical in experiments; however, traditional validation methods may lack sensitivity to hidden bias, such as distribution imbalance or non-linear interaction among participants. This study investigated the capability of supervised and unsupervised machine learning (ML) models to detect randomization flaws. Feature importance analyses were also conducted to identify predictors of potential assignment bias. A series of actions were carried out, including participant recruitment, implementation of a well-randomized sample assignment, training of various machine learning models, and evaluation of model performance on a binary classification task using accuracy, precision, recall, and F1-score. In this study, 12 participants were randomly assigned to play a learning direction game in two interface sequences (2D3D or 3D2D).

### Supervised vs. unsupervised models

All supervised ML models, logistic regression, decision trees, and SVM, achieved satisfactory classification performance (67%) when trained on labeled data (Bichri et al., 2024; Zhang et al., 2017). After incorporating synthetic data, their performance further improved, with all three models reaching up to 87% accuracy. The unsupervised ML model k-means achieved only 58%. KNN and ANN consistently plateaued at 67%, even after synthetic data were added. The ANN model showed the weakest performance, with precision as low as 44%, likely due to the small sample size and its limited ability to capture meaningful patterns in binary classification (Alloghani et al., 2019; Alwosheel et al., 2018; Pudjihartono et al., 2022). While synthetic data improved ANN performance, it also introduced overfitting, as indicated by inflated precision scores. Overall, supervised ML models achieved higher accuracy than unsupervised models in detecting randomization flaws, thereby supporting H1.

### Feature importance and assignment bias

Feature importance analysis offers a practical means of guiding unsupervised ML models by identifying variables that most strongly influence cluster separation (Pudjihartono et al., 2022). In this study, feature importance results revealed consistent findings across models. In both KNN and ANN, the total scores earned in the 2D game and the total time spent collecting all treasures in the 2D game emerged as key predictors. In k-means clustering, the time spent on the sixth treasure hunt in the 3D game (No. 18, Table 1) was identified as an important feature. Gender and gameplay frequency were consistently less important, although gender showed slightly greater predictive relevance. This suggests that the experimental design achieved effective randomization with respect to demographic variables. At the same time, the findings indicate that assigning participants to groups based on gameplay performance risks data leakage, as learners may be prematurely categorized as low or high performers. Overall, the evidence supports H2: Feature importance analysis reveals key predictors of assignment bias.

### Confirmation of randomization and learning outcomes

The randomization procedures implemented in this study reflect a systematic sample assignment process. The results demonstrated that supervised ML classifiers effectively validated the randomization in binary classification, confirming that the two experimental treatments produced distinct learning outcomes in both scores and gameplay times. With supervised ML validation, the differences in learning outcomes (higher scores and faster completion in the 2D-first condition) can be attributed to treatment effects rather than pre-existing biases. Conversely, if independent variables such as gender or gaming frequency had significantly influenced the outcomes, supervised ML classifiers would have performed only at chance level (~50% accuracy), providing neither support for randomization nor evidence of model sensitivity. Unsupervised ML classifiers, which are designed to detect latent patterns and predictive relationships, may in some cases outperform supervised approaches in classification tasks. However, this was not the case in the present study. Overall, the consistent performance across multiple ML methods confirmed both the success of the randomization procedure and the impact of interface sequence on learning outcomes.

### Learning performance in 2D vs. 3D

Participants assigned to the 2D3D sequence achieved higher scores than those in the 3D2D sequence. This aligns with expectations, as 2D representations are inherently simpler and less cognitive demanding than 3D representations (Herbert and Chen, 2015; Hegarty, 2011; Hicks et al., 2003). Two-dimensional tasks allow learners to concentrate more effectively on core learning concepts without the distraction of additional spatial complexity. In contrast, 3D representations requires greater cognitive effort to interpret and are less prevalent in traditional educational materials. Cognitive science research further supports that 2D visualizations demand less mental effort and are more easily processed by the visual system than 3D representations (Chang et al., 2017; Hegarty, 2011). The human visual system processes 2D flat images more easily because all elements are presented on a single plane. This reduces the cognitive load and enhances comprehension. Consequently, 2D representations tend to be more accessible for learners due to their simplicity, familiarity, ease of application, and reduced cognitive demand. Nevertheless, combining 2D and 3D perspectives can be beneficial in certain domains such as ML model development (Chen et al., 2019) and structural design (Hong et al., 2024).

### Implications for game-based learning

Game-based learning is gaining increasing popularity across educational levels and age groups (Liu et al., 2020; Sumi and Sato, 2022). Beyond the complexity of 3D games, the literature emphasizes their immersive effects of 3D games, particularly in virtual reality (VR) and augmented reality (AR) settings, compared to traditional 2D games. Learners frequently report greater excitement and engagement with 3D games than with 2D ones (Chang et al., 2017; Dalgarno and Lee, 2010). To ensure that learners achieve their learning objectives, it is important to incorporate virtual guides or an instructional pages that present fundamental subject concepts within game environment (Chiu et al., 2022; Dicheva et al., 2021; Yilmaz and Cagiltay, 2016). Recent advances in intelligent learning companions, powered by well-trained machine learning models, further enhance the educational value of game-based learning environments. The development of intelligent learning companions, powered by well-trained ML models, has advanced significantly. These companions provide personalized interaction and adaptive support during gameplay, thereby improving engagement and learning outcomes (Alloghani et al., 2019; Alwosheel et al., 2018; Pudjihartono et al., 2022).

### Limitations

While ML shows promise for validating randomization, its reliability depends strongly on sample size and experimental context. As shown in the ANN results, performance was poorest with the limited sample size (*n* = 12). After synthetic data were added, overfitting emerged, suggesting that the model failed to generalize. In this study, the direction game served as the experimental scenario. Players required some adaptation to orient themselves within the environment before gameplay. This was especially challenging in the 3D2D treatment, which relied heavily on spatial orientation skills. Once players adapted to the 3D environment, however, performance improved more rapidly in the 2D3D treatment, where participants transitioned from simpler to more complex tasks. If the game is either too easy or too difficult, causing players to consistently achieve full scores or perform poorly, the resulting learning outcomes become highly predictable. In such cases, sample randomization would be trivial, and ML classification would add little value because no meaningful patterns could be explored. Therefore, the findings of the present study should be regarded as preliminary. They may not easily generalize to larger or more complex experimental designs, where greater variability in participant performance and richer datasets could lead to more robust validation of randomization.

## Conclusion

This study contributes to advancing the use of machine learning (ML) models for validating sample randomization in experimental assignments. A carefully designed recruitment plan, followed by a well-randomized sample assignment, was shown to be enabling the evaluation of ML performance. Supervised ML models (i.e., logistic regression, decision trees, and SVM) achieved a satisfactory level of accuracy in detecting randomization validity. Feature importance analysis further demonstrated that while ML offers considerable promise, its reliability is contingent on factors such as sample size, noise tolerance, and experimental context.

Compared with traditional validation methods, ML models can capture complex and subtle biases, offering a more sensitive evaluation of non-linear relationships and higher-order interactions. In this study, ML models were employed as a robustness check to validate experimental randomization during sample assignment. The novelty lies in demonstrating that ML can be used to evaluate claims of participant randomization in experimental designs, suggesting its potential as a supplementary tool. However, while ML shows promise in detecting randomization patterns, its efficacy depends on sample size and design complexity. With very small samples, its reliability remains limited. Future work should therefore apply this approach to larger and more balanced datasets, combining ML with traditional balance tests (e.g., *t*-test, *F*-test). It is also recommended that ML be systematically compared with standard balance tests across diverse experimental contexts.

Finally, future studies are encouraged to extend this framework to experiments with more than two treatments. The capabilities of other ML models, including semi-supervised and self-supervised approaches, should also be explored in classification tasks to further expand understanding in this area.

## Data Availability

The raw data supporting the conclusions of this article will be made available by the author, without undue reservation.
